# An Optimal Deep Hybrid Framework with Selective Kernel U-Net for Skin Lesion Detection and Classification

**DOI:** 10.3390/bioengineering13040427

**Published:** 2026-04-06

**Authors:** Guzal Gulmirzaeva, Robert Hudec, Baxtiyorjon Akbaraliev, Batirbek Samandarov

**Affiliations:** 1 Department of Multimedia and Information-Communication Technology, University of Žilina, 010 01 Žilina, Slovakia; robert.hudec@uniza.sk; 2 Department of Systematic and Practical Programming, Tashkent University of Information Technologies Named After Muhammad Al-Khwarizmi, Amir Temur Avenue 108, Tashkent 100084, Uzbekistan; botirbek_s@mamunedu.uz; 3 Andijan State University, Andijan 170100, Uzbekistan; b.akbaraliev@adu.uz; 4 Department of General Professional Sciences, Mamun University, Bolkhovuz Street 2, Khiva 220900, Uzbekistan

**Keywords:** skin cancer detection, dermoscopic images, SK-UNet segmentation, Fossa Optimization Algorithm, deep learning, feature selection

## Abstract

Early and accurate detection of skin cancer is critical for reducing mortality rates, particularly for malignant melanoma. Automated analysis of dermoscopic images has gained significant attention due to its potential to support clinical diagnosis and overcome the limitations of manual inspection. Motivated by challenges such as image noise, low contrast, lesion variability, and redundant feature representation, this study proposes an optimal deep hybrid framework for skin lesion detection and classification. The objective of this work is to design a robust and efficient system that integrates advanced preprocessing, precise segmentation, optimal feature selection, and accurate classification. Initially, contrast enhancement using Contrast Limited Adaptive Histogram Equalization (CLAHE) and noise reduction using Wiener filtering are applied to improve image quality. Lesion regions are then segmented using a Selective Kernel U-Net (SK-UNet), which adaptively captures multi-scale spatial information. Subsequently, discriminative color, texture, and shape features are extracted and optimized using the Fossa Optimization Algorithm (FOA) to eliminate redundancy. A hybrid one-dimensional Convolutional Neural Network–Gated Recurrent Unit (1D-CNN–GRU) classifier is employed for final classification, learning both spatial and sequential feature patterns. Experimental evaluation on the ISIC and DermMNIST datasets demonstrates that the proposed framework achieves classification accuracies of 97.6% and 95.6%, respectively, outperforming several existing methods. The results confirm that the proposed hybrid framework provides reliable, accurate, and scalable skin cancer diagnosis, highlighting its potential for assisting clinical decision-making and early detection.

## 1. Introduction

**Figure 1:**
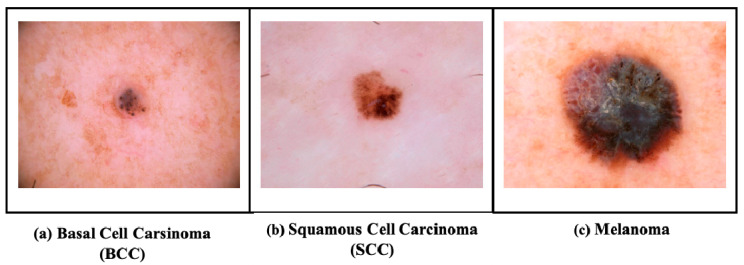
Dermoscopic images of common skin cancer types obtained from the ISIC Archive (https://www.isic-archive.com/, accessed on 15 May 2025).

Millions of people are affected by skin cancer (SC), which is becoming more common in the world because of environmental and other reasons [1]. It consists of three major forms: basal cell carcinoma (BCC) which develops gradually with no or minimal metastatic potential but is capable of causing local tissue damage [2]; squamous cell carcinoma (SCC), which is more aggressive and can manifest itself on parts of the body exposed to the sun; and lastly, the most uncommon form but quite lethal, melanoma, which is formed by melanocytes with high metastatic ability and hence early detection and treatment are essential [3,4]. Figure 1 presents the common types of SCs.

Melanoma is a condition that develops due to the unregulated enlargement of malignant melanocytes and could also spread very fast unless it is diagnosed early [5]. The U.S. has a reported rate of 106,110 new melanoma cases and 7180 melanoma-related deaths in 2021, and a worldwide non-melanoma SC incidence rate of 2–3 million per year, and a melanoma-related incidence rate of 132,000 cases each year [6] and [7] respectively. The main cause of SC is DNA mutation due to long-term exposure to ultraviolet (UV) radiation [2], along with other risk factors such as tanning beds, immune deficiency, and an inherited tendency. The deadliest SC is melanoma because it is aggressive and can metastasize in a short span of time [8].

Skin texture and color can be influenced by other skin conditions, including infections, allergies, or chronic diseases and predisposition to cancer [9,10]. SCs that are malignant should be detected in the early stages because untimely diagnosis may prove to be fatal [11,12]. Routine screening and community awareness of self-examination would help reduce late diagnoses and improve outcomes.

Machine learning (ML) and DL, as subdivisions of Artificial Intelligence (AI), have also contributed to dermatology diagnostics to a considerable extent [4], alleviating the shortage of qualified dermatologists by providing independent and accurate support [13]. DL, particularly CNNs, derive hierarchical features of DIs to detect melanoma at an early stage [11], and Explainable AI (XAI) enhances transparency and trust. Screenings based on dermoscopic and standard digital images provide personalized healthcare and can reduce skin cancer mortality through low-cost, accessible screening with no requirement for manual ROI selection [14]. Innovative technologies open the path to customized healthcare solutions, enabling SC to be detected and treated more effectively at early stages, thereby reducing mortality rates globally.

### 1.1. Research Motivation

SC is considered one of the most rapidly increasing forms of cancer across the globe, with melanoma being the most lethal form of cancer. The success of treatment relies on early and accurate diagnosis, but the primary limitation of conventional manual dermoscopic diagnosis is subjective, slow, and prone to error. Current computer-aided systems are limited by image noise, lesion variability, and poor generalization. To overcome these issues, a hybrid DL framework that combines state-of-the-art segmentation, effective feature extraction, and optimization-based feature selection is suggested to provide a reliable and cost-effective SC diagnostic framework.

### 1.2. Objective of the Study

The primary objective of this study is to develop an accurate and robust hybrid DL framework for automated SC detection and classification using dermoscopic images. Specifically, the study aims to enhance image quality through effective preprocessing, achieve precise lesion segmentation using SK-UNet, extract and optimize discriminative color, texture, and shape features using the FOA, and perform reliable multi-class classification using a hybrid 1D-CNN–GRU model. The proposed framework seeks to improve diagnostic accuracy, robustness, and generalization across the benchmark ISIC and DermMNIST datasets.

### 1.3. Research Contribution

The significant findings of the current work will be as follows:

- **Advanced Preprocessing**: A combination of CLAHE and Wiener filtering of DIs is applied to enhance contrast, reduce noise, and provide uniform image quality.
- **Precise Segmentation**: SK-UNet is used to segment lesion areas, enabling precise boundary detection regardless of lesion shape or size.
- **Rich Feature Extraction**: A variety of complementary features are obtained, including color (RGB and HSV), texture (GLCM, LBP), and shape (roundness, saturation, dispersity), ensuring that the lesion is comprehensively represented.
- **Optimized Feature Selection**: FOA selects the most informative features, reduces redundancy, and enhances the classifier’s efficiency.
- **Hybrid Classification Model**: A hybrid 1D-CNNGRU model is developed to integrate spatial feature learning with sequential dependency modeling, resulting in robust multi-class classification performance.

### 1.4. Paper Organization

The structure of the paper is as follows: Section 2 is a review of related SC studies; Section 3 outlines the proposed hybrid framework; Section 4 presents the results of testing and comparisons; Section 5 discusses the contributions and limitations; and future study objectives are concluded in Section 6.

## 2. Literature Review

Recent advances in DL have significantly improved the accuracy and robustness of SC and skin lesion classification systems. Kyi Pyar Zaw and Atar Mon [15] proposed an ensemble-based framework integrating Inception-V3, ResNet-50, and VGG-16 architectures to classify multiple types of skin lesions using the ISIC dataset. Their approach effectively categorized lesions such as melanoma, basal cell carcinoma (BCC), and squamous cell carcinoma (SCC), demonstrating the strength of ensemble learning in dermatological image analysis.

Faheem Mazhar et al. [16] introduced a CNN-based SC detection framework optimized using the Gray Wolf Optimization (GWO) algorithm. The methodology includes preprocessing, segmentation, feature extraction, and classification stages, in which CNNs extract discriminative features from regions of interest, thereby improving diagnostic accuracy.

Saima Ali Batool et al. [17] presented a hierarchical DL model using transfer learning for skin lesion diagnosis. Two CNN architectures were employed to classify seven types of nevi using the HAM10000 dermoscopic image dataset, achieving improved classification performance through multi-level decision-making.

Eatedal Alabdulkreem et al. [18] developed a lightweight DL framework combining pre-trained models such as GoogleNet, ResNet-18, and MobileNetV2 with a custom lightweight CNN (LWCNN). The model was trained on Kaggle datasets to classify skin images into melanoma and non-melanoma categories while reducing computational complexity.

Sankarakutti Palanichamy Manikandan et al. [19] proposed an Efficient DL Classification System (EDLCS) that emphasizes color feature analysis for melanoma detection. The study evaluated multiple color spaces, including RGB, HSI, and LAB, and highlighted the importance of color information for accurate melanoma diagnosis.

Muhammad Mateen et al. [20] introduced a hybrid DL framework combining U-Net for segmentation, InceptionResNet-V2 for feature extraction, and a Vision Transformer with self-attention for feature refinement. Hyperparameter optimization further enhanced early and precise SC classification performance.

Ahmad Naeem et al. [21] proposed SNC-Net, which integrates handcrafted features with DL-based feature extraction to improve classification efficiency. The model was trained and validated using dermoscopic images from the ISIC 2019 [22,23,24] dataset, demonstrating strong performance in SC diagnosis.

Mohammed Yousif et al. [25] presented a hybrid framework that combines a binary Gray Wolf Optimization algorithm for feature selection with CNN-based classification. The optimization process effectively reduced redundant features, lowered computational cost, and improved classification accuracy.

Khadija Nawaz et al. [26] proposed a Feature-Constrained Data-Specific CNN (FCDS-CNN) architecture to address class imbalance and enhance data quality in melanoma datasets. The model significantly improved lesion detection accuracy through data-specific feature learning.

Mohammed Alshahrani et al. [27] developed a hybrid system combining Random Forests and Feed-Forward Neural Networks to extract complex features from dermoscopic images, leveraging the strengths of both traditional ML and DL methods.

Recently, Subhayan Mukherjee et al. [28] introduced an explainable DL framework for SC detection using a Swish-activated deep CNN. The use of the Swish activation function improved feature learning capability while enhancing model interpretability, addressing the transparency limitations of conventional CNN-based approaches.

Furthermore, Bibhuprasad Sahu et al. [29] proposed an ensemble transfer learning approach coupled with the Osprey Optimization Algorithm for optimal feature selection from SC image datasets. The optimization-driven feature selection significantly improved classification accuracy while reducing dimensionality and computational overhead. A review of the existing approaches for SC detection and classification is presented in Table 1.

**Table 1:** Review of approaches for SC detection and classification.

Ref. No	Author Details	Techniques	Advantages	Disadvantages
[15]	Kyi Pyar Zaw and Atar Mon	VGG-16, ResNet 50, InceptionV3	Faster, robust, and scalable for clinical use	May reduce accuracy, add complexity, and need more resources
[16]	Faheem Mazhar et al.	CNN, GWO	Fast, accurate, accessible, and reliable diagnosis.	High cost, data limits, and complexity.
[17]	Saima Ali Batool et al.	CNN	High accuracy, better recall, and effective early detection.	High computation needs and poor generalization on imbalanced data.
[18]	Eatedal Alabdulkreem et al.	GoogleNet, ResNet-18, and MobilNet-v2	Higher accuracy and better early detection with preprocessing.	Computationally intensive and slower execution.
[19]	Sankarakutti Palanichamy Manikandan et al.	EDLCS	High accuracy with effective noise removal and color models.	Limited to binary classification and dataset dependency.
[20]	Muhammad Mateen et al.	Hybrid DL	Highly accurate, generalizable, reduces misdiagnosis, and app-friendly.	Complex, resource-heavy, dataset-dependent, and deployment challenges
[21]	Ahmad Naeem et al.	SNC_Net	Accurate, multi-class, balanced, and explainable.	Limited scope, complex, less generalizable, and incomplete
[25]	Ahmad Naeem et al.	Hybrid CNN and GWO	Reliable and fast melanoma diagnosis.	High computing demands and limited dataset testing
[26]	Khadija Nawaz et al.	FCDS-CNN	Accurate and dependable early detection of skin cancer	Requires significant computing resources and quality data.
[27]	Mohammed Alshahrani et al.	CNN models	Reliable and precise early skin cancer diagnosis	Computationally complex and resource-intensive

## 3. Proposed Approach

**Figure 2:**
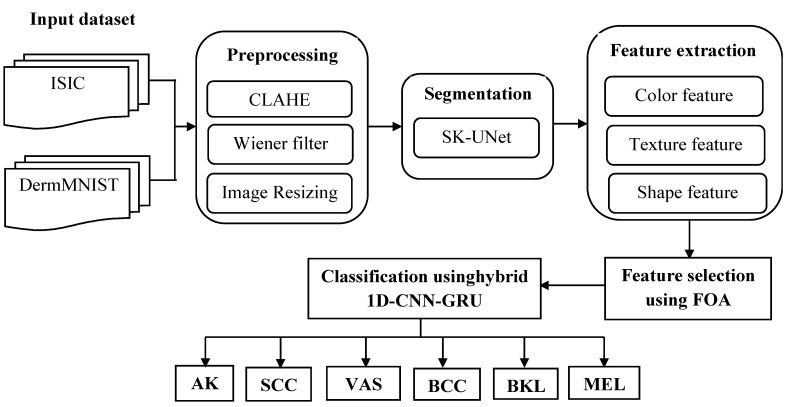
The complete steps of the proposed methodology for skin cancer detection and classification.

Melanoma, an extremely severe type of SC, requires early and precise diagnosis, and this investigation suggests a novel hybrid DL technique for supervised melanoma detection and classification using DIs. The model is evaluated on ISIC and DermMNIST datasets, with input images preprocessed using CLAHE for contrast enhancement, Wiener filtering for noise reduction, and resizing for uniform dimensions. Preprocessed images are segmented with SK-UNet, and features are extracted from lesions, including color (RGB, HSV), texture (GLCM, LBP), and shape (dispersity, saturation, roundness). Then, FOA selects the most informative features, and a hybrid 1D-CNN-GRU model performs classification, capturing spatial and sequential patterns for accurate SC detection. The overall workflow of the proposed SC detection and classification framework is illustrated in Figure 2. A detailed explanation of each component is provided in the following sections, and the mathematical formulations of the preprocessing and segmentation stages are presented in Equations (1)–(10).

### 3.1. Dataset Collection

The proposed model uses two benchmark dermoscopic image datasets for training and assessment:

- **ISIC and DermMNIST datasets**

**Figure 3:**
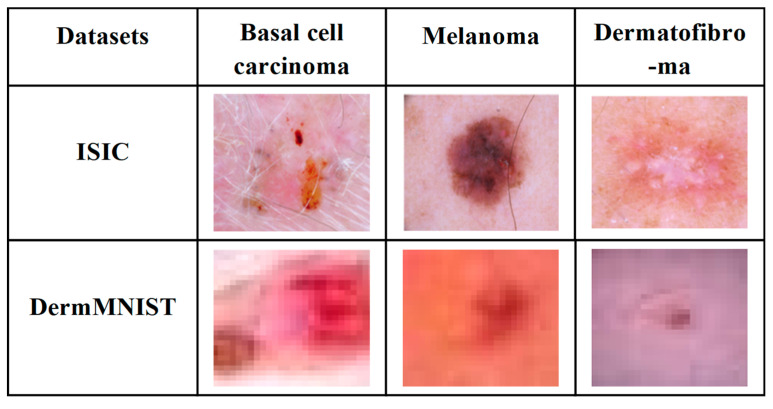
Sample input images from the ISIC and DermMNIST datasets.

The ISIC and DermMNIST datasets are widely used benchmarks for SL analysis and melanoma detection. The ISIC dataset provides high-quality DIs with annotated lesion regions across multiple SL classes, ensuring a diverse set of lesion types for training and testing models, and is publicly available at ISIC Dataset https://www.kaggle.com/datasets/nodoubttome/skin-cancer9-classesisic (accessed on 15 May 2025). Similarly, the DermMNIST dataset offers standardized, annotated dermoscopic images for lesion classification tasks, supporting the evaluation of model generalizability, and can be accessed at DermMNIST Dataset https://www.kaggle.com/datasets/dayanandshettar/dermamnist (accessed on 15 May 2025). Figure 3 illustrates sample dermoscopic images from the ISIC and DermMNIST datasets used for training and evaluation.

### 3.2. Data Preprocessing

**Figure 4:**
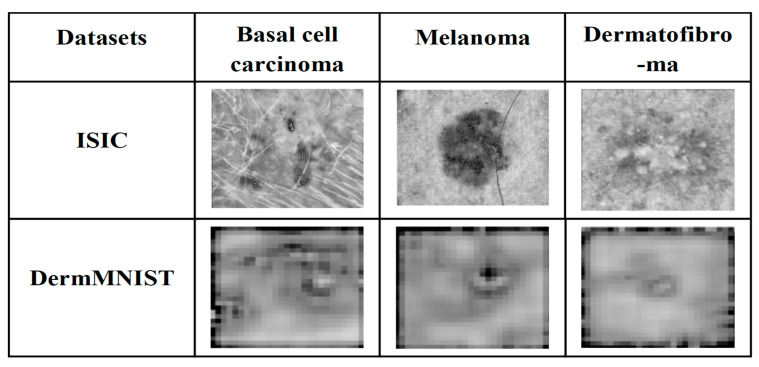
Preprocessed output images from the ISIC and DermMNIST datasets.

Preprocessing is essential in DI analysis to enhance the clarity of the images, reduce noise, and prepare data for classification. To address issues like low contrast, illumination variations, and artifacts, this work applies three techniques: CLAHE for contrast enhancement, the Wiener filter for noise reduction, and resizing for standardizing image dimensions. Figure 4 shows the preprocessed output images after applying CLAHE and Wiener filtering, highlighting improved contrast and noise suppression.

#### 3.2.1. CLAHE

CLAHE is an image enhancement technique that improves local contrast while minimizing noise amplification. It divides the image into pixels in order to apply histogram normalization to each, thereby enhancing features that might be lost due to uneven lighting or inadequate contrast. The following provides a detailed description of the steps required to implement this method:

✓ **Set Clip Limit and Region Size:** The process is initiated by setting the clip limit and region size for each region using the histogram shape. A histogram’s clip limit can be calculated using Equation (1):
  (1) $$\beta =\frac{R}{g}(1+\phi *100*(S_{Max}+1))$$
  where g is the highest grayscale value (256 for an 8-bit image), R is the size of the region (tile) for which the histogram will be produced, $M_{Max}$ is the maximum pixel value in the region, and ϕ is the clip factor (specified between 0 and 100), which controls the level of histogram clipping [30].
✓ **Histogram Clipping:** Each tile’s histogram is clipped using the clip limit (β) to make sure that no pixel intensity goes over the specified limit. This step stops noise from being amplified too much.
✓ **Excess Redistribution:** A new histogram is created and applied to the relevant tile in the image by redistributing any excess from the clipped histogram—that is, the pixel values that surpass the limit—across the region.
✓ **Final Image Construction:** The final image is produced by smoothing down the borders, enhancing the local contrast, and interpolating pixel values from nearby regions [31].

#### 3.2.2. Wiener Filter

Wiener filtering is primarily used to filter a picture based on the accuracy of the image noise variance estimation, meaning that after calculating the local mean and variance values of the pixels, the mean square error between the original image $P\left(m,n\right)$ and $q\left(m,n\right)$, the filtered image must be reduced. ϖ and η stand for the negative weld image’s width and height, respectively, centered on $\left(m,n\right)$ [32]. The pixel gray value’s mean and variance are computed as follows:

(2) $$\mu =\frac{1}{\varpi \times \eta }\sum_{m,n}^{\varpi ,\eta }P\left(m,n\right)$$

(3) $$\sigma^{2}=\frac{1}{\varpi \times \eta }\sum_{m,n}^{\varpi ,\eta }{\left(P\left(m,n\right)-\mu \right)}^{2}$$

where $\sigma^{2}$ represents the variance value of the intended processed pixel point and μ represents the mean value of the pixel point that needs processing [32]. The mean square error is then:

(4) $$\xi =\frac{1}{\varpi \times \eta }\sum_{m,n}^{\varpi ,\eta }\mu \left(m,n\right)\sigma^{2}\left(m,n\right)$$

The Wiener filter formula is as follows:

(5) $$Q\left(m,n\right)=\mu \left(m,n\right)+\xi \left(P\left(m,n\right)-\mu \left(m,n\right)\right)$$

#### 3.2.3. Image Resizing

Image resizing uses upsampling or downsampling to change the image’s proportions to fit the specifications of the model or display. While bilinear and bicubic interpolation provide smoother results, the nearest neighbor approach is quick but may result in blocky artifacts. To guarantee effective processing and preserve significant features, all of the photos in this study have been resized to 224 × 224 pixels, which is a standard DL size [33].

The above techniques are utilized to preprocess the input images, and the enhanced outputs are then forwarded to the segmentation stage for further analysis.

### 3.3. Segmentation Using SK-UNet

**Figure 5:**
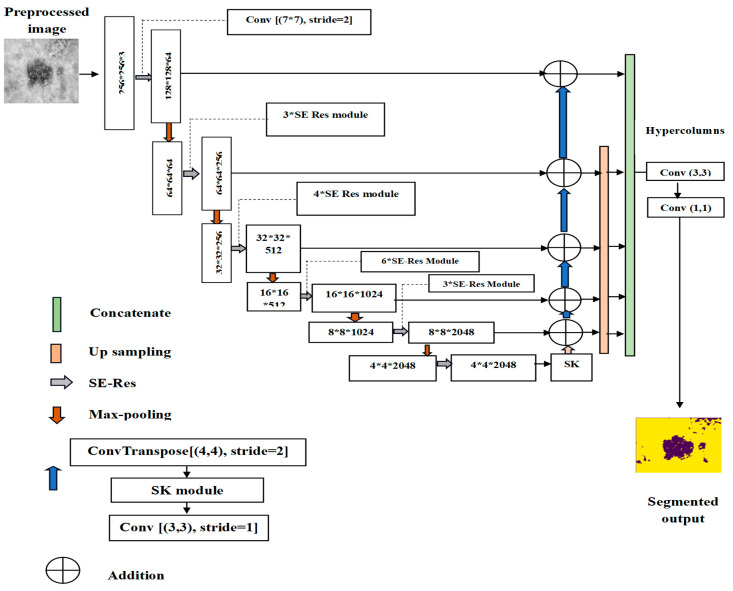
Framework of the proposed SK U-Net for segmentation.

Segmentation is essential to the perception of medicine analysis by isolating regions of interest, such as lesions or tumors. In this work, SK-UNet is used for precise SL segmentation. As an enhanced version of U-Net, SK-UNet adaptively adjusts its receptive fields to capture multi-scale features and fine lesion boundaries. Its dynamic kernel selection enables accurate detection of irregular shapes, asymmetry, and subtle structures, outperforming the traditional U-Net with fixed kernels. Figure 5 depicts the architectural framework of the proposed SK-UNet model employed for accurate lesion segmentation [34].

In the SK U-Net methodology, an SK module is added in the decoder, and a squeeze-and-excitation residual (SE-Res) module is included in the encoder. The detailed explanation of each process is shown below,

➢ **Squeeze-And-Excitation Residual (SE-Res) Module**

The SK U-Net encoder has six spatial resolution stages, with downsampling via two max-pooling layers and three 1 × 1 stride-2 convolutions. At each stage, feature transformation uses three convolutions (1 × 1, 3 × 3, 1 × 1) with batch normalization, ReLU, and an SE-Res module. The SE-Res module compresses geographical data globally into channel descriptors and generates channel weights, enhancing the network’s feature representation.

➢ **Selective Kernel (SK) Module**

The SK U-Net decoder reconstructs the segmentation map through multiple upsampling steps using adaptive transposed convolutions. At each stage, the feature map is processed with BN, ReLU, and convolution, followed by an SK module to capture multi-scale information. The input feature map O is split into three branches with different dilated convolutions, producing $F^{\prime }$, $F^{\prime \prime }$, and $F^{\prime \prime \prime }$, which are summed element-wise to form the integrated feature map F. Global average pooling of F generates channel-wise information $g\in \beta^{c}$, and a fully connected layer produces the aggregated weight vector $U\in \beta^{D\times 1}$. Adaptive attention weights $U^{\prime }$ are then computed for each branch to learn multi-scale spatial features.

(6) $$U^{\prime }=W⨂UU^{\prime }\epsilon \beta^{3c}$$

where ⨂ represents the 1 × 1 convolution operation and matrix W stands for the acceptable characteristics in the fully connected layer. It is possible to create a new weight matrix that includes three rows ($P^{\prime }$, $Q^{\prime }$, and $R^{\prime }$) and R columns by reshaping $U^{\prime }$. The weight matrix is further transformed using softmax attention to produce probability weighting components for every channel, as shown by the subsequent formulas.

(7) $$p_{m}=\frac{\vartheta^{P_{m}}}{\vartheta^{P_{m}}+\vartheta^{Q_{m}}+\vartheta^{R_{m}}}$$

(8) $$q_{m}=\frac{\vartheta^{Q_{m}}}{\vartheta^{P_{m}}+\vartheta^{Q_{m}}+\vartheta^{R_{m}}}$$

(9) $$r_{m}=\frac{\vartheta^{R_{m}}}{\vartheta^{P_{m}}+\vartheta^{Q_{m}}+\vartheta^{R_{m}}}$$

In the $m^{th}$ channel, the learnable parameters are represented by the symbols $p_{m}$, $q_{m}$, and $r_{m}$. The final weighted feature map is computed by:

(10) $$\hat{O_{i}}=\sum p_{m}F_{m}^{\prime }+q_{m}F_{m}^{\prime \prime }+r_{m}F_{m}^{\prime \prime \prime }$$

**Figure 6:**
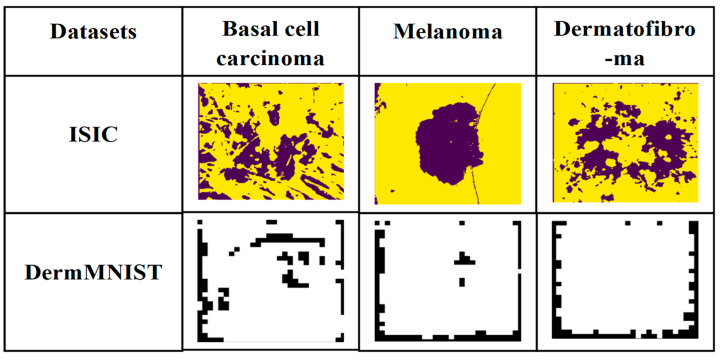
Segmented output images from the ISIC and DermMNIST datasets. The lesion regions are shown in dark/purple, while the background is shown in yellow/light color.

In the output feature map $\hat{O_{i}}$, the $m^{th}$ feature channel is indicated by the notation $p_{m}+q_{m}+r_{m}=1$, where $\hat{O_{i}}$ is defined. With the help of these learnable parameters, the network can automatically adapt and change itself to find the best weights for feature maps filtered by various kernel sizes. After the decoder, feature maps are upsampled to the input image’s resolution and concatenated. The aggregated map undergoes 1 × 1 convolution, BN, ReLU, and another 1 × 1 convolution, with softmax applied to produce class probability values for the final segmentation map. This combination of SK and SE-Res modules permits the network to obtain both global contextual information and fine-grained lesion details, producing highly accurate segmentation masks [35]. Figure 6 shows the segmentation output images using SK U-Net.

The above-described selective kernel U-Net (SK U-Net) is employed to divide the preprocessed images, and the resulting segmented outputs are then passed to the feature extraction stage for further analysis.

### 3.4. Feature Extraction

The feature extraction step transforms the regions of segmented lesions into useful numerical features to be used in classification. It records important lesion features, color, texture, and shape, to be able to distinguish between cases and identify minor changes in patterns. Three feature categories are employed in this work, which are color features (RGB and HSV), texture features (GLCM and LBP), and shape features (dispersity, saturation and roundness).

#### 3.4.1. Color Features Using RGB and HSV

Color characteristics present crucial information regarding the patterns of pigmentation and color variations in lesions, which are crucial in separating between normal and malignant regions.

✓ **RGB**

Images are analyzed by the RGB color space, which consists of red, green, and blue channels. The mean and standard deviation of the RGB channels have been determined to represent the color change on a particular image block. Red channel is more vivid and green, and blue is overlapped. Such figures are utilized to discern among SC types.

✓ **HSV**

The HSV color model splits hue (H), saturation (S), and value (V) to have a type of color, purity, and brightness. Although H has extensive distribution and great contrast, on its own it cannot differentiate between normal or neoplastic skin. On the other hand, the V component is quite effective in pointing out irregular pigmentation and boundary differences and is a favored tool in the process of identifying melanoma and other lesions of skin cancer [36].

#### 3.4.2. Texture Feature Using GLCM and LBP

The texture characteristics are used to detect the architecture and intensity differences in lesions, and this allows the identification of abnormalities that are a sign of malignancy.

✓ **GLCM**

The texture characterization is based on the analysis of variations in the surface patterns of an image, and the GLCM technique is employed to obtain six central characteristics of Angular Second Matrix (ASM), Contrast, Similarity, Homogeneity, Energy, and Correlation, which exist at different gray levels and angles. The characteristics are useful in identifying mild structural variations in SLs, which can be used to differentiate between benign and malignant ones.

The difference between the luminance of one pixel compared to another and the subsequent one is referred to as contrast, and this is used to quantify the change in intensity in a picture. They are calculated mathematically as follows:

(11) $$contrast=\sum_{m}\sum_{n}\rho \left(m,n\right)*{\left\lceil m-n\right\rceil }^{2}$$

The degree to which pairs of pixels in an image with different gray levels may be distinguished from one another is measured by dissimilarity. This is exactly what it is computed:

(12) $$dissimilarity=\sum_{m}\sum_{n}\rho \left(m,n\right)*\left\lceil m-n\right\rceil$$

The homogeneity of the gray-level distribution and texture thickness inside an image are measured by the angular second moment. Here are ways to calculate this metric:

(13) $$asm=\sum_{m}\sum_{n}\rho {\left(m,n\right)}^{2}$$

The extent of variation in the textual components of the image, especially in terms of uniformity, is measured by homogeneity. The formula below is used to compute it:

(14) $$homogeneity=\sum_{m}\sum_{n}\frac{\rho \left(m,n\right)}{1\;+{\;\left(m,n\right)}^{2}}$$

The stability of gray-level changes in an image’s texture is measured by energy. The energy calculation looks like this:

(15) $$energy=\sum_{m}\sum_{n}\rho (m,n)$$

Correlation measures whether an image’s grayscale values are arranged in rows or columns. The correlation calculation appears as follows:

(16) $$correlation=\sum_{m}\sum_{n}\frac{\left(m\;-\;mean\right)*\left(n\;-\;mean\right)\;*\;\rho {\left(m,n\right)}^{2}}{variance}$$

The variables “m” and “n,” which stand for the coordinates of the image’s pixels on the m-axis and n-axis in the GLCM, are used to calculate the texture characteristics. ρ(m,n) shows the quantity of pixels at a particular location. The “mean” refers to the average value in the texture feature, whereas the “variance” indicates the dispersion or spread of the values in the texture feature [37].

✓ **Local Binary Pattern (LBP)**

LBP is a simple, efficient, and rotation- and scale-invariant texture descriptor that encodes local image texture by comparing neighboring pixel intensities to the central pixel within a 3 × 3 window, assigning binary values accordingly (Equation (17)).

(17) $$LBP(X,Y)=\left\{\begin{array}{c} 1,\;if\;\sigma \geq 0\\ 0,\;otherwise \end{array}\right.$$

where LBP(X,Y) indicates the pixel intensity. After computation, the LBP texture descriptor’s histogram yields 256 values. The N × N blocks are used to calculate the histogram. The LBP histogram aids in achieving the rotation-invariant and scale-invariant nature by minimizing the feature vector length [38].

#### 3.4.3. Shape Feature Using Dispersion, Saturation, and Roundness

Shape features capture the geometric structure of lesions, using metrics such as area (Λ) and perimeter (ρ) (Equations (18)–(20)), along with dispersion, saturation, and roundness, to analyze boundary irregularities and distinguish cancerous regions.

✓ Dispersion measures the spread of pixels in a region, calculated as the ratio of the lesion area to the square of its perimeter. The following is the dispersion calculation:
  (18) $$dispersity=\frac{\rho^{2}}{\Lambda }$$
✓ Convexity (or saturation) measures how closely a region’s shape resembles a convex form, calculated as the ratio of its area to perimeter. The following formula is used to determine saturation (or convexity):
  (19) $$saturation=\frac{\Lambda }{\rho }$$
✓ Circularity (or roundness) indicates the degree to which a region resembles a circle [37]. The following is the circularity calculation:
  (20) $$roundness=\frac{\left(4\;*\;\Pi *\;\Lambda \right)}{\rho^{2}}$$

The above techniques are employed to extract aspects of shape, color, and texture from the lesion regions that have been segmented. After that, the features that were extracted are sent to the feature selection stage for further processing.

### 3.5. Feature Selection Using FOA

In high-dimensional datasets, redundant or irrelevant features can reduce classifier performance and increase computational cost. To address this, the proposed framework employs FOA for efficient feature selection, aiming to retain informative features and improve accuracy. FOA is inspired by the fossa’s hunting behavior, consisting of: (i) attacking the lemur’s observed location and (ii) pursuing the lemur across trees. Unlike conventional feature selection methods, which may get trapped in local optima or fail to balance exploration and exploitation, FOA provides a better balance between exploration and exploitation due to its bio-inspired search strategy, which helps avoid local optima and select highly discriminative features.

✓ **Initialization**

In the proposed FOA-based feature selection method, the initialization stage begins with the random generation of candidate solutions, where each solution represents a subset of features. Each fossa agent $S_{m}$ is a potential subset of features. A binary vector with a length equivalent to the entire assortment of features is used to encode the subset in the dataset. Within this vector, each element, 1. $s_{m,D}=1$ the $D^{th}$ feature is selected and 2. $s_{m,D}=0$ the $D^{th}$ feature is not selected. The initial population matrix is expressed as:

(21) $$S={\left[\begin{array}{c} S_{1}\\ \vdots \\ S_{m}\\ \vdots \\ S_{R} \end{array}\right]}_{R\times i}=\left[\begin{array}{c} s_{1,1}\ldots s_{1,D}\ldots s_{1,i}\\ \vdots \\ s_{m,1}\ldots s_{m,D}\ldots s_{m,i}\\ \vdots \\ s_{R,1}\ldots s_{R,D}\ldots s_{R,i} \end{array}\right]$$

where $S_{m}$ indicates the $m^{th}$ fossa, representing a feature subset.$\;s_{m,D}\in \left\{1,0\right\}$ is the selection status of the $D^{th}$ feature in the $m^{th}$ subset. R is the number of fossas, i is the quantity of decision variables, equal to the quantity of features. The initialization of each feature decision variable is performed as:

(22) $$s_{m,D}=\cap_{D}+a\cdot (\cup_{D}-\cap_{D})$$

In this setup, a there is a random number in [0, 1], $\cap_{D}$ $\cup_{D}$ are the lower and upper bounds of the $D^{th}$ feature, set as $\cup_{D}=1$ and $\cap_{D}=0$ for feature selection. Initialization generates diverse random feature subsets $S_{m}$, each evaluated using an objective function that balances classification accuracy and subset size, aiming to optimize predictive performance while reducing dimensionality.

(23) $$fu={\left[\begin{array}{c} {fu}_{1}\\ \vdots \\ {fu}_{m}\\ \vdots \\ {fu}_{R} \end{array}\right]}_{R\times 1}={\left[\begin{array}{c} {fu(S}_{1})\\ \vdots \\ {fu(S}_{m})\\ \vdots \\ {fu(S}_{R}) \end{array}\right]}_{R\times 1}$$

In this instance, fu stands for the vector of evaluated objective function values, and ${fu}_{m}$ denotes the objective function value that corresponds to the $m^{th}$ fossa, representing the quality of its selected feature subset.

✓ **Fitness Function**

To guide the search towards optimal feature subsets, a fitness function is used that balances classification accuracy and subset size reduction. The fitness of a feature subset $S_{m}$ is defined as:

(24) Fitness=Max(Accuracy)

✓ **Mathematical modeling for FOA**

The FOA was created by mimicking the natural fossa’s clever movement patterns. Two separate stages are used to update the FOA members’ locations within the problem-solving space:

1. **Exploration Phase:**

This phase is inspired by a fossa’s early attack behavior when hunting a lemur, focusing on extensively exploring the search space to identify potential areas. The changes in the fossa’s position as it prepares and launches its attack are used to guide the positional updates in the algorithm.

2. **Exploitation Phase:**

In this phase, the fossa fine-tunes its approach to focus on the target, simulating the lemur chase. The algorithm exploits promising areas by updating positions based on the fossa’s dynamic movements during pursuit.

Below is a full description and mathematical modeling of each update phase in FOA.

✓ **Attacking and moving towards the lemur**

In the first FOA phase (attack phase), population members update their positions by mimicking a fossa chasing a lemur, enhancing global exploration. Potential lemur locations are determined by comparing objective function values, as described in Equation (25).

(25) $${cl}_{m}=\left\{S_{K}:{fu}_{K}<{fu}_{m}\;and\;K\neq m\right\},\;where\;m=1,2,\ldots ,R\;and\;K\in \{1,2,\ldots R\}$$

The set of possible lemur locations for the $m^{th}$ fossa is represented by ${cl}_{m}$ In this equation, $S_{K}$ indicates which member of the population has a higher median function level than the $m^{th}$ fossa, and the corresponding objective function value is ${fu}_{K}$. FOA assumes a random lemur is attacked, with each member’s new position calculated using Equation (26) and replacing the previous position if it yields a higher objective function value, as per Equation (27).

(26) $$s_{m,n}^{\rho 1}=s_{m,n}+a_{m,n}\cdot (\psi_{m,n}-\zeta_{m,n}\cdot s_{m,n})$$

(27) $$S_{m}=\left\{\begin{array}{c} S_{m}^{\rho 1},\;{fu}_{m}^{\rho 1}<{fu}_{m},\\ S_{m},\;else, \end{array}\right.$$

Here, $\psi_{m}$ indicates the lemur selected by the $m^{th}$ fossa, and $\psi_{m,n}$ indicates the $n^{th}$ dimension of this lemur’s position. With *x*$S_{m}^{\rho 1}$ being its $n^{th}$ dimension, *XiP*1 denotes the newly calculated position for the $m^{th}$ fossa during the attack phase of the FOA. At this new point, the objective function value is ${fu}_{m}^{\rho 1}$. Random values between 0 and 1 are denoted by the words $a_{m,n}$, and random numbers between 1 and 2 are denoted by $\zeta_{m,n}$.

✓ **Chasing to catch a lemur**

In the second phase of FOA, the fossa’s chase of the lemur updates population positions to enhance local search and exploitation. Small positional changes simulate the natural pursuit, with each member’s new position calculated using Equation (28) and replacing the previous position if it yields a higher objective function value, as per Equation (29).

(28) $$s_{m,n}^{\rho 2}=s_{m,n}+(1-2a_{m,n})\cdot \frac{\cup_{n}-\cap_{n}}{\iota }$$

(29) $$S_{m}=\left\{\begin{array}{c} S_{m}^{\rho 2},\;{fu}_{m}^{\rho 2}<{fu}_{m},\\ S_{m},\;else, \end{array}\right.$$

In this case, the updated position calculated for the $m^{th}$ fossa during the chasing phase in the proposed FOA is represented by $S_{m}^{\rho 2}$. The $n^{th}$ dimension of this new position is shown by each $s_{m,n}^{\rho 2}$, and the accompanying objective function value is indicated by ${fu}_{m}^{\rho 2}$. The current iteration count is denoted by ι, whereas the variables $a_{m,n}$ are random values between [0, 1].

✓ **Termination**

FOA iteratively updates candidate solutions until a stopping requirement is satisfied and the best-performing solution at termination symbolizes the optimal feature subset [39]. The FOA is utilized to identify the most relevant features, and the optimal feature subset obtained from the best solution is then forwarded to the classification phase.

### 3.6. Classification Using Hybrid 1D-CNN-GRU

In this stage, SLs are classified using selected handcrafted features through a hybrid 1D-CNN–GRU model. Although the features are not temporal, they are arranged as an ordered one-dimensional sequence based on diagnostic relevance. This ordering reflects the typical dermatological analysis process. The GRU is used to model inter-feature dependencies and contextual relationships rather than temporal information, enabling the network to capture complex feature correlations and improve classification accuracy.

✓ **Input Layer:**

The vector of chosen features that represent the properties of SLs serves as the model’s input and is provided to the model as a 1D sequence.

✓ **1D-CNN Layer**

The 1D-CNN layer automatically extracts local correlations and patterns from the feature sequence. Since the features are sequential (1D), the convolution kernel moves in one direction, capturing distinct patterns from the data [40]. Multiple convolution kernels are applied to detect various types of discriminative information relevant to skin cancer detection.

✓ **Batchnorm Layer**

Batch normalization is applied after the 1D-CNN layer to stabilize and accelerate training, reduce overfitting, and handle common DL issues like gradient explosion or vanishing.

(30) $$C_{\vartheta }=\frac{C-\eta }{\sqrt{\in^{2}-\xi }}\lambda +\phi$$

where C is the vector data output of the previous layer, ϑ is the batch normalization η, and ${s\in }^{2}$ stands for mean and variance, respectively, and ξ is a constant added to the variance for data stability. Hence, the batch normalization equation states that λ it ϕ will acquire factors for resizing and adjusting the normalized values.

✓ **GRU layer**

The GRU layer captures temporal and sequential dependencies in the feature sequence. GRU is chosen over LSTM for its simpler structure, faster training, and fewer parameters. The GRU processes the data using the following steps:

**Update gate:** The quantity of data that was saved from the previous instant’s memory to the current time step. The equation is:

(31) $$U_{\tau }=\sigma (\omega_{U}\cdot s_{\tau }+X_{U}\cdot H_{\tau -1}+B_{U})$$

**Reset gate:** The state of the preceding instant controls the way information is used to determine the candidate state. The formula is:

(32) $$R_{\tau }=\sigma (\omega_{R}s_{\tau }+X_{R}\cdot H_{\tau -1}+B_{R})$$

**Update candidate hidden state:** Determine the candidate’s concealed state by utilizing the reset gate data. The formula is:

(33) $$E_{\tau }=tanH(\omega_{E}s_{\tau }+X_{E}(R_{\tau }⊙H_{\tau -1})+B_{E})$$

**Update the current state:** Contains two pieces of information: the candidate’s hidden state as of right now and the state as of the last second. The formula is:

(34) $$H_{\tau }=\left(1-U_{\tau }\right)⊙E_{\tau }+U_{\tau }⊙H_{\tau -1}$$

In this case, σ stands for the sigmoid activation function, tanH for the hyperbolic tangent activation function, ω for the weights, $U_{\tau }$ for the update gate, $R_{\tau }$ for the reset gate, $E_{\tau }$ for the candidate hidden state, $H_{\tau }$ for the final hidden state, and ⊙ for the element-by-element product, the input τ at time step $s_{\tau }$ [41].

✓ **Fully Connected Layer and Softmax Classifier**

The flattened GRU output is sent to a fully connected layer, which converts the recovered features into the resulting classes. The results are transformed into probability using the softmax function:

(35) $$Softmax\left(s_{m}\right)=\frac{e^{s_{m}}}{\sum_{n=1}^{N}e^{s_{n}}}$$

where $s_{m}$ is the input value and N is the number of categories to be classified.

✓ **Output Layer**

**Figure 7:**
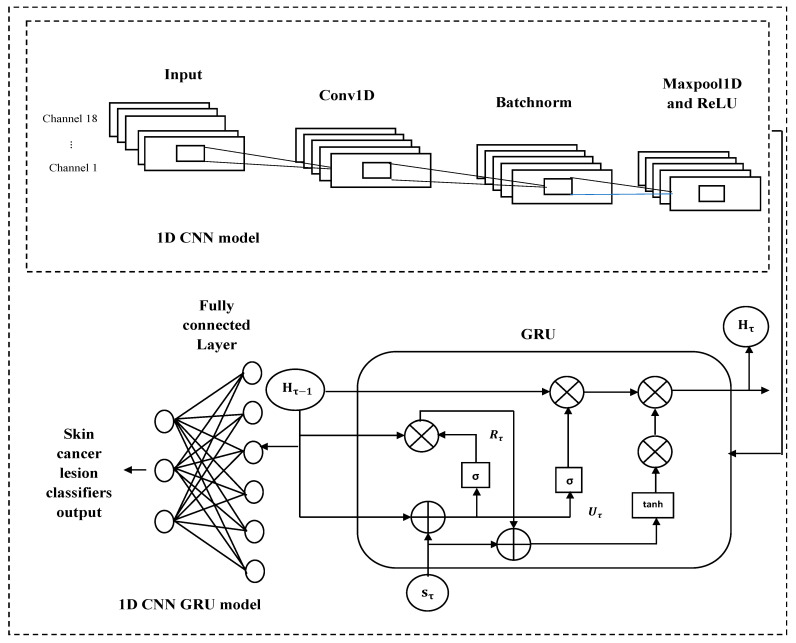
Hybrid 1D-CNN-GRU architecture.

The predicted skin cancer type is determined as the most likely classification from the softmax output, providing the final classification result, as shown in Figure 7 [42].

By utilizing the hybrid 1D-CNN–GRU architectural design, the proposed model effectively captures both local feature representations and long-term sequential dependencies, enabling accurate classification of SC. The overall workflow of the proposed SC detection and classification framework is summarized in Algorithm 1, which presents the step-by-step execution of the system. The algorithm outlines image acquisition, preprocessing, lesion segmentation using SK-UNet, feature extraction, FOA-based feature selection, and final classification using the hybrid 1D-CNN–GRU model.
**Algorithm 1:** Proposed SK-UNet + FOA + Hybrid 1D-CNN–GRU Framework
**Input:** Dermoscopic images from ISIC and DermMNIST datasets **Output:** Classified skin lesion type **Step 1:** Acquire dermoscopic images from benchmark datasets. **Step 2:** Apply preprocessing using:    (a) CLAHE for contrast enhancement     (b) Wiener filtering for noise reduction    (c) Image resizing to 224 × 224 **Step 3:** Segment lesion regions using SK-UNet. **Step 4:** Extract features from segmented lesions:    (a) Color features (RGB, HSV)    (b) Texture features (GLCM, LBP)    (c) Shape features (dispersion, saturation, roundness) **Step 5:** Apply FOA to select optimal features. **Step 6:** Feed selected features into the hybrid 1D-CNN–GRU classifier. **Step 7:** Perform classification using softmax activation. **Step 8:** Evaluate performance using accuracy, precision, recall, F1-score, and AUC.

To ensure transparency and reproducibility, the proposed framework operates under the following assumptions: (I) all dermoscopic images are preprocessed using identical CLAHE and Wiener filtering parameters to maintain uniform image quality; (II) lesion segmentation accuracy directly influences feature reliability, hence SK-UNet is employed to minimize boundary errors; (III) the FOA selects features by jointly optimizing classification accuracy and feature subset size; and (IV) the hybrid 1D-CNN–GRU classifier assumes sequential dependency among selected handcrafted features. These assumptions are validated through extensive experimental analysis and ablation studies. The following section presents the experimental evaluation conducted to confirm the performance and efficacy of the suggested approach.

## 4. Results

The proposed SCD and classification framework was implemented using Python 3.x. Experimental evaluation was carried out on the ISIC and DermMNIST datasets. All experiments were conducted on a system equipped with an Intel Core i7 processor, 16 GB RAM, and an NVIDIA GPU with 8 GB memory. The models were trained using the Adam optimizer with a learning rate of 0.0001 and a batch size of 32 for 30 epochs. The datasets were divided into training (70%), validation (15%), and testing (15%) subsets. Model performance was assessed using accuracy, precision, recall, F1-score, and area under the curve (AUC) metrics for both datasets. Evaluation was performed for each lesion class, including Melanoma (MEL), Basal Cell Carcinoma (BCC), Actinic Keratosis (AK), Benign Keratosis (BKL), Dermatofibroma (DF), Nevus (NV), Pigmented Benign Keratosis (PBK), Seborrheic Keratosis (SK), Squamous Cell Carcinoma (SCC), and Vascular Lesion (VASC). To rigorously validate the effectiveness of the proposed hybrid framework, multiple evaluation strategies are adopted, including feature importance analysis, dimensionality-reduction visualization (t-SNE and PCA), confusion matrices, class-wise and overall performance metrics, ROC and Precision–Recall curves, and ablation studies. Furthermore, this section outlines the experimental findings and provides a comprehensive comparative performance analysis, with the results summarized in Table 2 and Table 3.

### 4.1. Evaluation of the Proposed Hybrid DL Classifier

This section assesses the suggested hybrid DL classifier using feature importance (FOA), t-SNE and PCA visualizations, confusion matrices, overall and class-wise performance metrics, training/validation accuracy and loss, ROC curves, and PR curves for both datasets, providing a comprehensive presentation of the results.

➢ **Proposed Feature Importance (FOA)**

**Figure 8:**
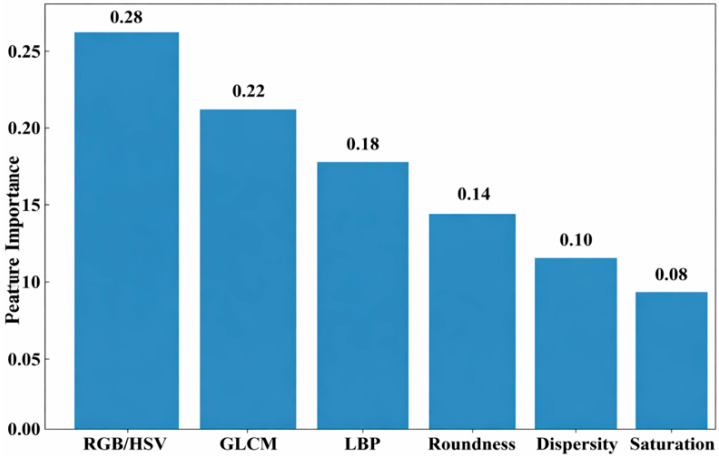
Feature importance analysis.

The characteristic importance for classifying skin cancer is displayed in Figure 8. Color features (RGB/HSV) have the highest importance (0.28), followed by texture features (GLCM = 0.22 and others = 0.18). Shape features—Roundness (0.14) and Dispersity (0.10)—have a moderate impact, while Saturation (0.08) contributes the least. Overall, color and texture features play the most significant roles in classification.

➢ **t-SNE and PCA feature visualization for both datasets**

**Figure 9:**
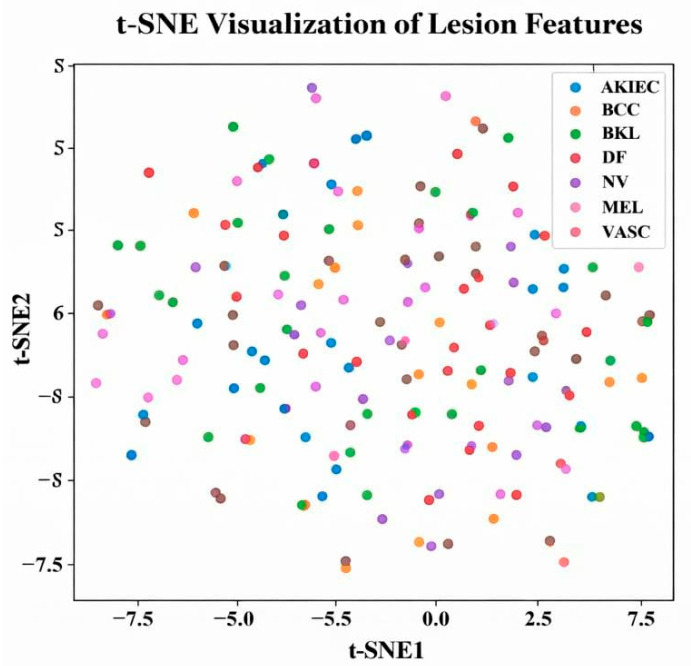
t-SNE visualization of lesion features.

Figure 9 displays the t-SNE visualization, which reduces high-dimensional lesion features to two dimensions for better class distribution understanding. Each point represents a lesion sample, with colors denoting classes (AKIEC, BCC, BKL, DF, NV, MEL, VASC). Although some overlap exists (e.g., NV, MEL, BKL), clear clustering patterns indicate t-SNE effectively captures non-linear separability among lesion features.

**Figure 10:**
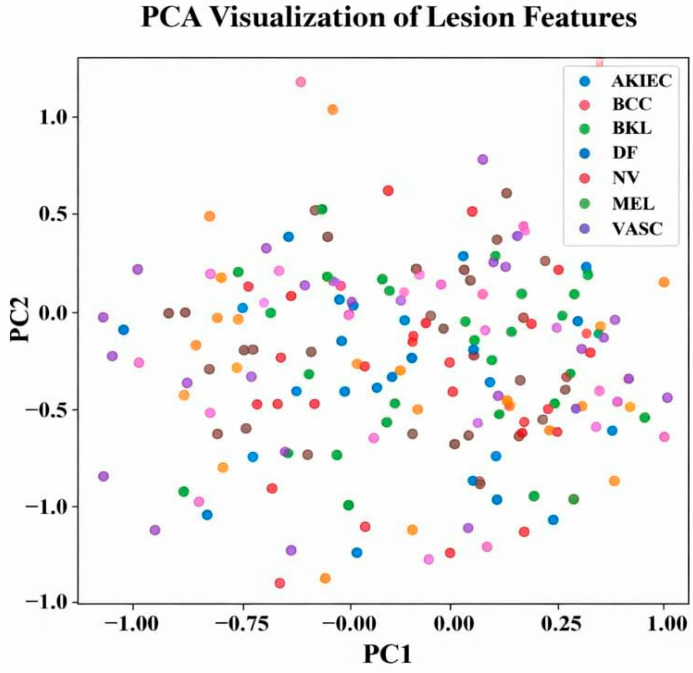
PCA visualization of lesion features.

Figure 10 presents the PCA visualization, projecting lesion features onto the first two principal components (PC1 and PC2) with values from −1.0 to +1.0. Classes (AKIEC, BCC, BKL, DF, NV, MEL, VASC) are color-coded. While some separation is visible, significant overlap—especially among NV, MEL, and BKL—shows the limitations of linear projection for distinguishing lesion types.

➢ **Confusion Matrix for both datasets**

A confusion matrix illustrates the categorization accuracy of a model by comparing real and predicted values. True Positives (TP), True Negatives (TN), False Positives (FP), and False Negatives (FN) are all included in binary classification. It extends into a grid for multi-class classification, with off-diagonal cells signifying incorrect classifications and diagonal cells representing accurate predictions.

**Figure 11:**
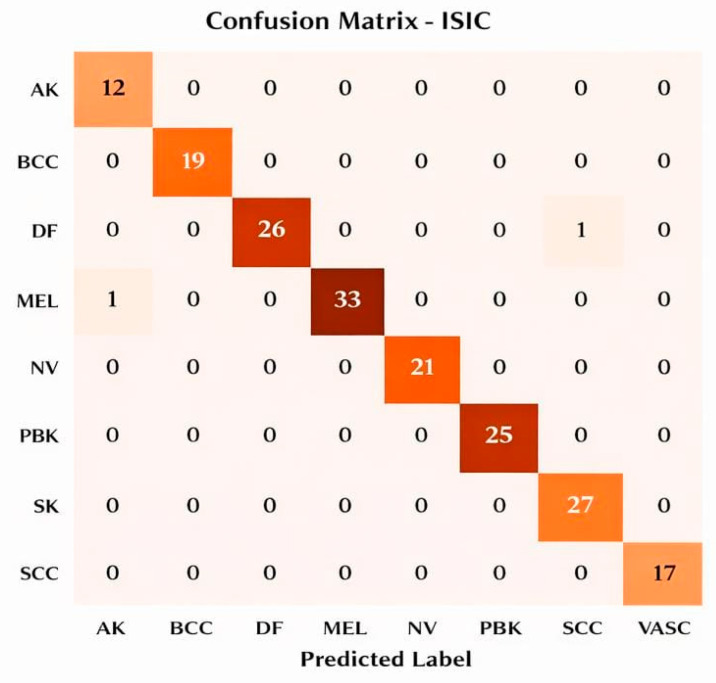
ISIC confusion matrix.

Figure 11 illustrates the confusion matrix for the ISIC dataset, demonstrating strong performance, with most instances correctly predicted along the diagonal. Misclassifications include one AK predicted as MEL, one DF predicted as SCC, one MEL predicted as DF, one MEL predicted as NV, and one NV predicted as PBK, with a total of 199 test instances.

**Figure 12:**
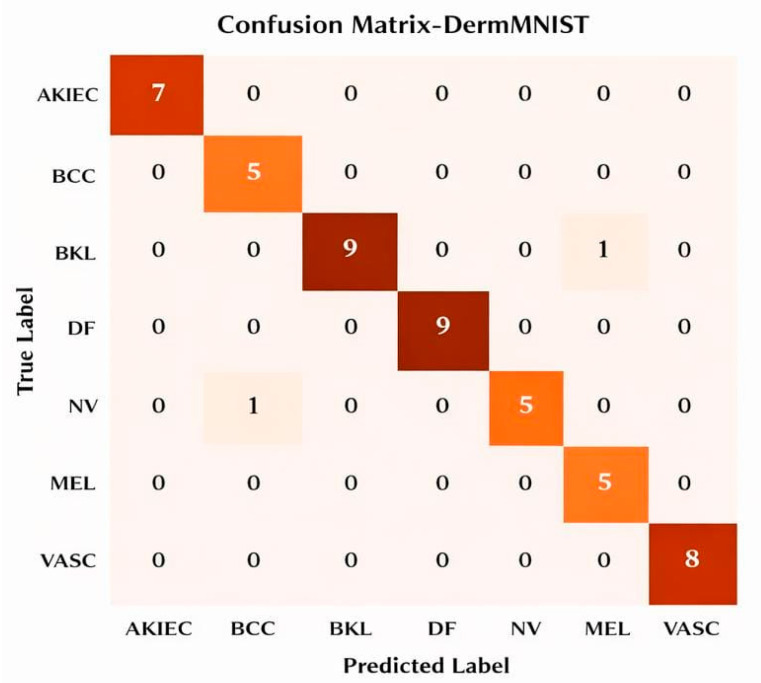
DermMNIST Confusion Matrix.

Figure 12 illustrates the confusion matrix for the DermMNIST dataset, in which the approach correctly classified most instances, with the majority of counts appearing on the diagonal: seven AKIEC, five BCC, nine DF, nine NV, five MEL, and eight VASC. The only misclassification observed is one BKL instance being predicted as MEL, out of a total of 49 test instances.

➢ **Performance Evaluation of the Proposed Hybrid DL Classifier on ISIC and DermMNIST Datasets**

A confusion matrix is utilized to assess the effectiveness of the model, offering a thorough examination of the proposed hybrid classifiers. The effectiveness and dependability of the suggested method are evaluated utilizing a range of achievement metrics; the outcomes of the proposed hybrid classifiers are compiled below.

The suggested hybrid classifiers performed well on a variety of criteria when tested on the ISIC and DermMNIST datasets. The model obtained 97.6% accuracy, 97.2% precision, 97% recall, 97.1% F1-score, and 98.5% AUC on the ISIC dataset. Likewise, it obtained 95.6% accuracy, 95.1% precision, 94.8% recall, 94.9% F1-score, and 96.8% AUC on the DermMNIST dataset. These results show that the hybrid classifiers excel in important measures while maintaining low error rates, maintaining high efficiency and consistency across both datasets.

➢ **Training and Validation Accuracy and Loss for both datasets**

**Figure 13:**
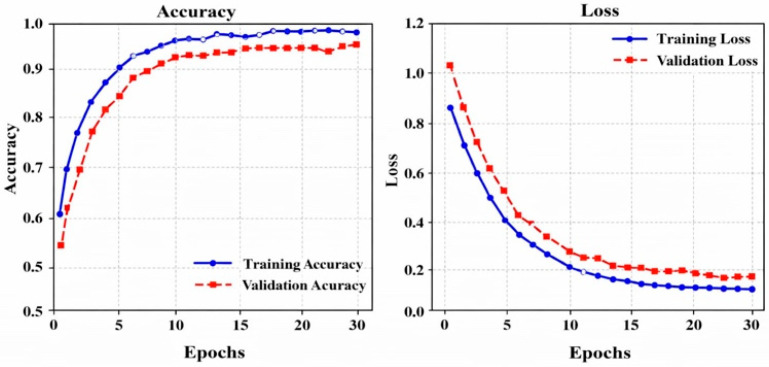
Training and Validation Performance over Accuracy and Loss for the ISIC dataset.

Figure 13 shows the DL model’s training over 30 epochs, with accuracy rising from 0.65 to 1.0 (training) and 0.67 to 0.98 (validation), while loss drops from 0.85 to 0.05 (training) and 1.0 to 0.15 (validation), indicating strong learning, convergence, and good generalization.

**Figure 14:**
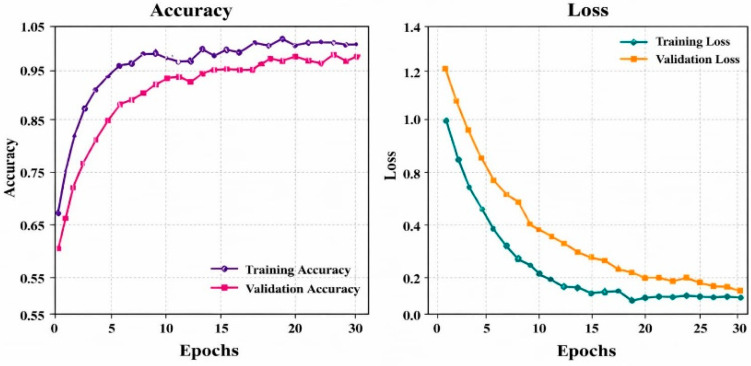
Training and validation performance over accuracy and loss for the DermMNIST dataset.

The model’s performance during 30 epochs of training and validation is displayed in Figure 14. While training loss decreases to 0.05 and validation loss decreases to 0.1, training accuracy increases from 0.65 to 0.96 and validation accuracy from 0.55 to around 0.95. The small gap between curves indicates steady convergence, efficient learning, and strong generalization with minimal overfitting.

➢ **Proposed performance metrics per class for both datasets**

**Figure 15:**
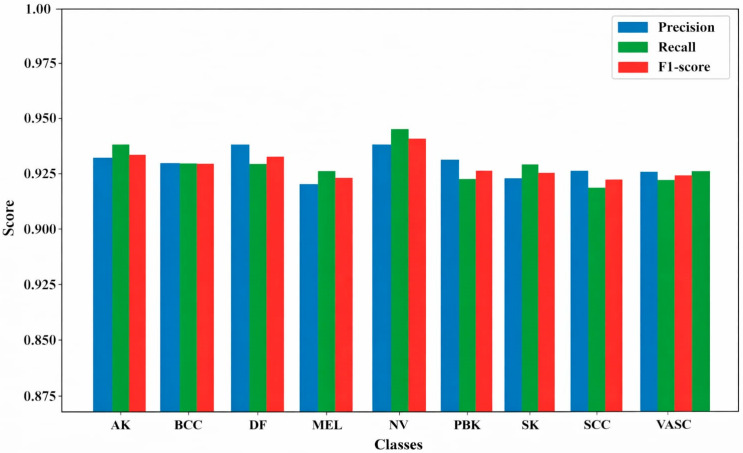
ISIC dataset classification performance per class.

Figure 15 demonstrates the model’s robust and reliable classification across SL classes. AK achieves a precision of 0.97 and a recall of 0.98, BCC has 0.97 each, DF reaches 0.98 precision and 0.97 recall, MEL shows 0.96 precision and 0.97 recall, NV performs best with 0.98 precision and 0.99 recall, PBK has 0.97 precision and 0.96 recall, SCC and SK range between 0.96 and 0.97 for both metrics, and VASC attains 0.97 each. Overall, the model exhibits high reliability and accuracy.

**Figure 16:**
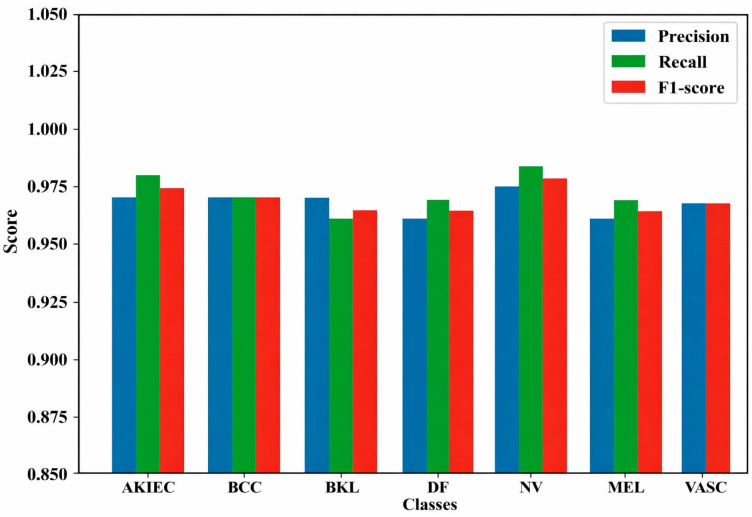
DermMNIST dataset classification performance per class.

Figure 16 shows that the model performs well consistently across all SL classes. AKIEC achieves precision 0.97 and recall 0.98, BCC has balanced precision and recall of 0.97, BKL attains an F1-score of 0.965 with precision 0.97 and recall 0.96, DF has precision 0.96 and recall 0.97, NV performs best with F1-score 0.985, precision 0.98, and recall 0.99, MEL shows precision 0.96 and recall 0.97, and VASC delivers balanced precision and recall of 0.97. Overall, the model demonstrates high reliability and accuracy in classifying different SLs.

➢ **Proposed ROC per class for both datasets**

**Figure 17:**
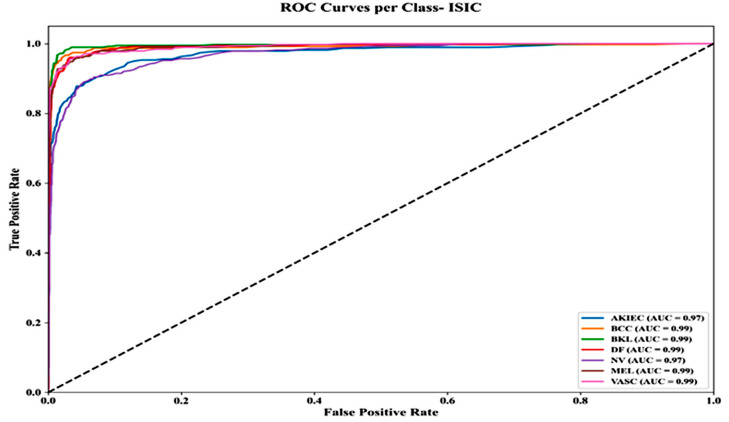
ROC curves per class—ISIC dataset.

Figure 17 shows ROC curves for all nine ISIC SL classes, indicating excellent classification performance. Most classes achieve AUC values of 0.99 (BCC, BKL, DF, PBK, VASC, MEL, SK), while AKIEC and NV reach 0.97. Curves rise quickly toward the top-left, with true positive rates > 0.95 and false positive rates < 0.05, demonstrating the model’s strong discrimination and minimal misclassification.

**Figure 18:**
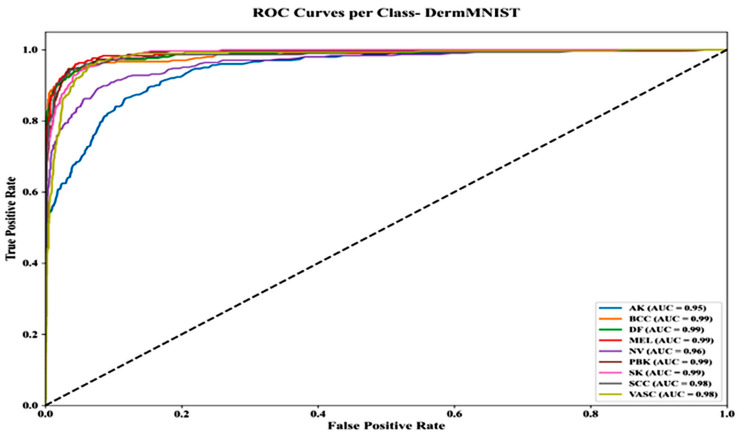
ROC curves per class—DermMNIST dataset.

Figure 18 shows ROC curves for the DermMNIST dataset, with most classes achieving high AUC values: BCC, DF, MEL, PBK, SK (0.99), SCC and VASC (0.98), NV (0.96), and AK (0.95). Curves demonstrate strong separation from the diagonal, with TP rates > 0.90 and FP rates < 0.10, indicating robust classification, though AK shows slightly lower performance.

➢ **Proposed PR curve per class for both datasets**

**Figure 19:**
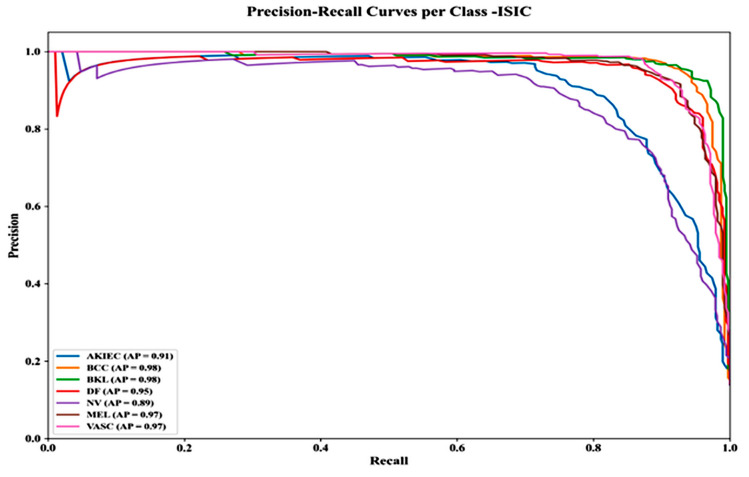
Precision–recall curves per class—ISIC dataset.

Figure 19 shows Precision–Recall curves for the ISIC dataset, highlighting strong performance across classes. Average Precision (AP) scores are: AKIEC 0.91, BCC 0.98, BKL 0.98, NV 0.89, MEL 0.97, VASC 0.97, and DF 0.95. Most classes maintain precision >0.95 up to 0.80 recall, demonstrating robust performance even for imbalanced data.

**Figure 20:**
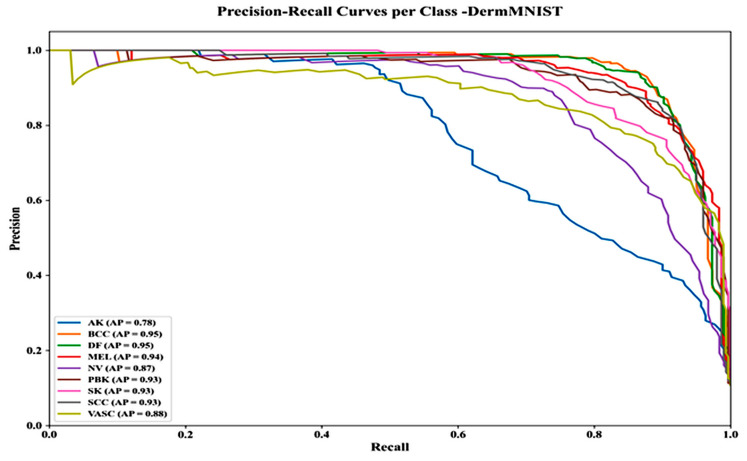
Precision–recall curves per class—DermMNIST dataset.

Figure 20 shows Precision–Recall curves for the DermMNIST dataset, revealing more variation across classes. AP scores are: SCC 0.93, SK 0.93, MEL 0.94, PBK 0.93, VASC 0.88, BCC 0.95, DF 0.95, NV 0.87, and AK 0.78. AK shows a notable precision drop after 0.40 recall, indicating greater classification challenges for some classes due to imbalance or complexity.

The detailed analysis of precision, recall, F1-score, ROC curves, and precision–recall curves on the ISIC and DermMNIST datasets demonstrates consistent and reliable classification performance across all lesion classes, confirming that the reported accuracy is not biased toward dominant classes.

### 4.2. Comparative Analysis for the Proposed Hybrid DL Model for Both Datasets

This section offers an illustration and evaluation of the suggested model and presents methods founded on performance metrics and an ablation study using the F1-score on ISIC and DermMNIST datasets. The comparison highlights the efficacy of the suggested approach, confirming its enhanced accuracy and reliability. The results obtained through the suggested methodology are illustrated in detail below, demonstrating its superior performance over baseline methods.

**Figure 21:**
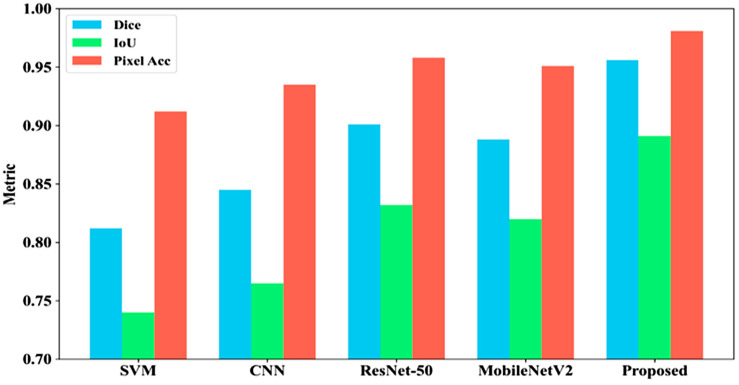
Comparative performance analysis of segmentation models for ISIC dataset.

Figure 21 compares five models for SL segmentation using Dice, IoU, and Pixel Accuracy. The proposed model leads with Dice 0.956, IoU 0.891, and Pixel Accuracy 0.981. ResNet-50 follows (0.901, 0.832, 0.958), MobileNetV2 shows moderate performance (0.888, 0.820, 0.951), CNN scores (0.845, 0.765, 0.935), and SVM performs worst (0.812, 0.740, 0.912), confirming the proposed model’s superior segmentation accuracy.

**Figure 22:**
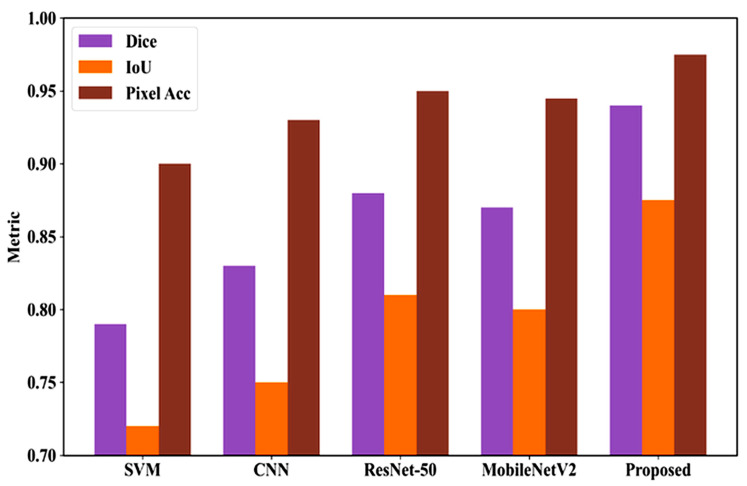
Comparative performance analysis of segmentation models for DermMNIST dataset.

Figure 22 compares models on the DermMNIST dataset for segmentation. SVM scores Dice 0.790, IoU 0.720, Pixel Accuracy 0.900; CNN improves to 0.830, 0.750, 0.930; MobileNetV2 achieves 0.870, 0.800, 0.945; ResNet-50 reaches 0.880, 0.810, 0.950. The proposed model outperforms all with Dice 0.940, IoU 0.875, and Pixel Accuracy 0.975, showing highly accurate lesion segmentation.

To validate feature selection, an experimental comparison of FOA with PCA, LASSO, GA, and PSO was conducted on the ISIC and DermMNIST datasets; Table 3 presents the obtained accuracy, precision, recall, and F1-scores for each feature selection method, highlighting FOA’s superior performance.

Table 3 shows that FOA outperforms traditional feature selection methods on the ISIC and DermMNIST datasets. Unlike PCA and LASSO, FOA effectively handles non-linear feature subsets, and unlike GA and PSO, it avoids premature convergence through its dual-phase exploration–exploitation strategy, ensuring selection of the most discriminative features.

**Figure 23:**
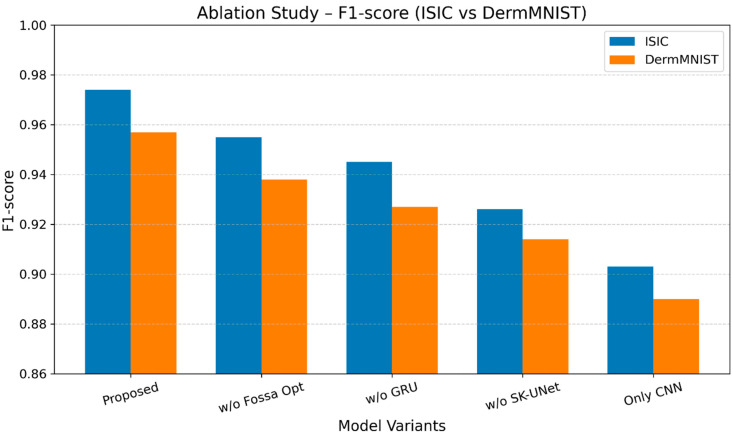
Ablation study F1-score performance analysis across ISIC and DermMNIST datasets.

Figure 23 shows the findings of the burning research on ISIC and DermMNIST datasets. The complete model achieves the highest F1-scores: 0.969 (ISIC) and 0.949 (DermMNIST). Removing Fossa optimization drops the scores to 0.946/0.927, GRU removal to 0.934/0.914, SK-UNet removal reduces them to 0.911/0.896, and using only CNN reduces them to 0.886/0.873. Results highlight the significant contributions of each component, with ISIC consistently showing higher F1-scores.

➢ **For the ISIC and DermMNIST datasets**

Table 4 presents a comprehensive comparative performance analysis of different classification models on the ISIC and DermMNIST datasets. The table clearly reports various metric values for each method, with dataset-wise separation to improve readability and interpretation. The proposed hybrid SK-UNet + FOA + 1D-CNN–GRU model demonstrates superior performance across all evaluation metrics when compared with baseline and state-of-the-art approaches.

Table 4 illustrates models on the ISIC and DermMNIST datasets using various metrics. The presented hybrid DL classifier outperforms baselines: on the ISIC dataset, it achieves 97.6% accuracy, 97.2% precision, 97% recall, 97.1% F1, and 98.5% AUC; on the DermMNIST dataset, 95.6% accuracy, 95.1% precision, 94.8% recall, 94.9% F1, and 96.8% AUC, demonstrating outstanding results in every metric. Results confirm that the proposed hybrid DL classifier outperforms existing SCD methods in accuracy, efficiency, and overall effectiveness compared to state-of-the-art approaches.

A comparison of the suggested hybrid DL approach with a number of previous studies is displayed in Table 5. The outcomes demonstrate that the suggested framework outperforms previously published techniques, achieving accuracies of 97.6% and 95.6% throughout the datasets. The suggested approach shows exceptional efficacy in SCD and classification by utilizing the power of hybrid DL classifiers.

## 5. Discussion

The proposed SCD and classification framework introduces a novel hybrid architecture that integrates SK-UNet-based adaptive segmentation, FOA-driven feature selection, and a hybrid 1D-CNN–GRU classifier to enhance diagnostic accuracy and robustness. The novelty of this study lies in the synergistic integration of adaptive multi-scale segmentation and bio-inspired feature optimization with sequential DL classification, which is rarely explored in existing skin lesion detection frameworks. Unlike conventional methods that rely on fixed kernel segmentation or direct end-to-end DL models, the proposed SK-UNet dynamically adjusts receptive fields to capture complex lesion boundaries, improving segmentation precision across lesions of varying sizes and shapes.

Furthermore, the incorporation of the FOA significantly improves feature selection by eliminating redundant and irrelevant lesion descriptors while preserving discriminative color, texture, and shape characteristics. This optimized feature representation reduces computational complexity and enhances classification efficiency. The hybrid 1D-CNN–GRU classifier further strengthens the framework by simultaneously learning spatial feature patterns and sequential dependencies, enabling more reliable classification of diverse SC types.

The significance of the proposed framework is demonstrated through superior performance across two benchmark datasets, achieving accuracies of 97.6% on the ISIC dataset and 95.6% on the DermMNIST dataset, outperforming several state-of-the-art deep learning and hybrid models. Additionally, the ablation study confirms that each component contributes substantially to overall system performance, validating the effectiveness of the integrated architecture. The framework provides a reliable and scalable solution for automated SC diagnosis, supporting early detection and clinical decision-making. However, challenges such as computational complexity, dependency on publicly available datasets, absence of explicit robustness evaluation, and the lack of clinical validation remain potential limitations. Consequently, the scope of the framework’s claims is limited to experimental performance on benchmark datasets. Future research will focus on dedicated robustness analyses under controlled noise, illumination variations, and lesion morphology perturbations, as well as validation on large-scale, real-world datasets, to further substantiate applicability. Additionally, the integration of explainable AI (XAI) techniques and optimization of model complexity for real-time deployment will be explored.

## 6. Conclusions

This study presented an effective hybrid DL framework for automated SCD and classification using dermoscopic images. The proposed approach integrates SK-UNet for accurate lesion segmentation, FOA for optimal feature selection, and a hybrid 1D-CNN–GRU classifier for robust multi-class classification. Experimental validation on the ISIC and DermMNIST datasets demonstrated high classification performance, achieving accuracies of 97.6% and 95.6%, respectively. The results confirm that the combination of precise segmentation, discriminative feature selection, and hybrid learning significantly enhances diagnostic accuracy and generalization across diverse lesion types.

The major strengths of the proposed framework include precise boundary detection through SK-UNet, reduced feature redundancy using FOA, and effective learning of both spatial and sequential patterns via the hybrid 1D-CNN–GRU classifier. The use of multiple benchmark datasets further demonstrates the robustness and reliability of the model. However, the framework also has certain limitations. It relies on publicly available datasets, which may not fully capture real-world clinical variability, and clinical validation has not yet been performed, limiting the generalizability of the results to actual scenarios. The computational complexity of DL and optimization components may also restrict real-time deployment on resource-constrained systems.

Future work will therefore focus on extending validation to large-scale datasets, performing explicit robustness evaluations under controlled noise, illumination variations, and lesion morphology perturbations, reducing computational complexity for real-time deployment, and integrating XAI techniques to enhance model transparency and clinician trust.

## Figures and Tables

**Table 2:** Performance analysis of proposed performance metrics.

Performance Metrics	Proposed	
	ISIC Dataset	DermMNIST Dataset
Accuracy	97.6%	95.6%
Precision	97.2%	95.1%
Recall	97%	94.8%
F-Measure	97.1%	94.9%
AUC	98.5%	96.8%

**Table 3:** Comparative performance of feature selection methods.

Feature Selection	ISIC Accuracy (%)	DermMNIST Accuracy (%)	ISIC F1-Score (%)	DermMNIST F1-Score (%)
PCA	94.1	91.8	93.7	91.4
LASSO	95.0	92.6	94.8	92.4
GA	96.1	93.9	95.9	93.5
PSO	96.4	94.2	96.2	94.0
FOA (Proposed)	97.6	95.6	97.1	94.9

**Table 4:** Evaluation of performance metrics in comparison for ISIC and DreamMNIST datasets.

Metrics	Dataset	SVM	CNN	ResNet	MobiliNetV2	Proposed
Accuracy	ISIC	86.4%	91.2%	94.5%	92.8%	97.6%
	Derm-MNIST	81.2%	87.6%	91.2%	89.4%	95.6%
Precision	ISIC	85.2%	90.8%	94.2%	92.1%	97.2%
	Derm-MNIST	80.5%	87%	90.7%	88.8%	95.1%
Recall	ISIC	84.7%	90.1%	93.9%	91.6%	97%
	Derm-MNIST	79.8%	86.4%	90.2%	88.2%	94.8%
F1-Score	ISIC	85%	90.4%	94%	91.8%	97.1%
	Derm-MNIST	80.1%	86.7%	90.4%	88.5%	94.9%
AUC	ISIC	88.1%	92.7%	95.6%	94.2%	98.5%
	Derm-MNIST	83.5%	88.9%	92.5%	91.2%	96.8%

**Table 5:** Performance comparison of the proposed hybrid DL classifier on ISIC and DermMNIST datasets against state-of-the-art published methods.

Ref. No	Techniques	Dataset	Accuracy (%)	Precision (%)	Recall (%)	F1-Score (%)	AUC (%)
[16]	CNN, GWO	HAM10000	95.11%	94.56%	93.88%	96.16%	-
[17]	CNN	HAM10000	97.4%	-	-	-	-
[26]	FCDS-CNN	melanoma dataset	96.66%	96%	96%	97%	96%
[43]	Intelligent Multilevel Thresholding with DL	ISIC	76.8%	69.3%	55.7%	60.6%	-
[44]	DenseNet-201+ Lasso+ Ensemble learning	ISIC	87.72%	-	92.15%	-	-
Proposed	SK-UNet + Fossa OA + Hybrid 1D-CNN-GRU	ISIC	97.6%	97.2%	97%	97.1%	98.5%
		DermMNIST	95.6%	95.1%	94.8%	94.9%	96.8%

## Data Availability

The data analyzed in this study are openly available from public sources. The ISIC dataset is available at the ISIC Challenge Datasets website https://challenge.isic-archive.com/data (accessed on 15 May 2025) and the DermaMNIST dataset is openly accessible on the MedMNIST platform and can be freely downloaded from https://medmnist.com (accessed on 15 May 2025).

## Abbreviations

The following abbreviations are used in this manuscript:

SC	Skin cancer
DL	Deep learning
CLAHE	Contrast Limited Adaptive Histogram Equalization
SK-UNet	Selective Kernel U-Net
FOA	Fossa Optimization Algorithm
1D CNN	One-dimensional Convolutional Neural Network
GRU	Gated Recurrent Unit
CNN	Convolutional Neural Network
BCC	basal cell carcinoma
SCC	squamous cell carcinoma
MEL	melanoma
AK	Actinic Keratosis
BKL	Benign Keratosis
DF	Dermatofibroma
NV	Nevus
PBK	Pigmented Benign Keratosis
SK	Seborrheic Keratosis
VASC	Vascular Lesion
AI	Artificial Intelligence
ML	machine learning
XAI	Explainable Artificial Intelligence
ROI	Region of Interest
SCD	Skin cancer Detection
GWO	Gray Wolf optimization
RF	Random Forest
SE Res	Squeeze-And-Excitation Residual
BN	batch normalization
ReLU	rectified linear unit
LBP	Local Binary Pattern
